# Development of response inhibition in the context of relevant versus irrelevant emotions

**DOI:** 10.3389/fpsyg.2013.00383

**Published:** 2013-07-02

**Authors:** Margot A. Schel, Eveline A. Crone

**Affiliations:** ^1^Institute of Psychology, Leiden UniversityLeiden, Netherlands; ^2^Leiden Institute for Brain and CognitionLeiden, Netherlands; ^3^Department of Psychology, University of AmsterdamAmsterdam, Netherlands

**Keywords:** response inhibition, development, emotion, adolescence, go/nogo task

## Abstract

The present study examined the influence of relevant and irrelevant emotions on response inhibition from childhood to early adulthood. Ninety-four participants between 6 and 25 years of age performed two go/nogo tasks with emotional faces (neutral, happy, and fearful) as stimuli. In one go/nogo task emotion formed a relevant dimension of the task and in the other go/nogo task emotion was irrelevant and participants had to respond to the color of the faces instead. A special feature of the latter task, in which emotion was irrelevant, was the inclusion of free choice trials, in which participants could freely decide between acting and inhibiting. Results showed a linear increase in response inhibition performance with increasing age both in relevant and irrelevant affective contexts. Relevant emotions had a pronounced influence on performance across age, whereas irrelevant emotions did not. Overall, participants made more false alarms on trials with fearful faces than happy faces, and happy faces were associated with better performance on go trials (higher percentage correct and faster RTs) than fearful faces. The latter effect was stronger for young children in terms of accuracy. Finally, during the free choice trials participants did not base their decisions on affective context, confirming that irrelevant emotions do not have a strong impact on inhibition. Together, these findings suggest that across development relevant affective context has a larger influence on response inhibition than irrelevant affective context. When emotions are relevant, a context of positive emotions is associated with better performance compared to a context with negative emotions, especially in young children.

## Introduction

The ability to cognitively control our actions is of critical importance for successful functioning. A well-studied component of cognitive control is response inhibition, which refers to the ability to refrain from a pre-potent response (Luna et al., 2010). Response inhibition has been shown to have a protracted developmental trajectory (van der molen, 2000; Bunge et al., 2002; Durston et al., 2002), with mature performance levels being reached in mid to late adolescence (Luna et al., 2010).

In daily life, cognitive control processes seldom stand-alone; one is often required to exercise cognitive control in a social or affective context. Sometimes the context can be relevant, for example, when you want to approach friendly people but inhibit from approaching unfriendly people, and sometimes context is irrelevant, for example when you want to approach people belonging to a certain group and inhibit from approaching people belonging to another group, independent of whether they are friendly or unfriendly. To examine the role of affective context on response inhibition, prior studies made use of an emotional go/nogo task (e.g., Tottenham et al., 2011). In this task, participants are instructed to respond to a certain emotional stimulus and inhibit responding to another emotional stimulus (such as emotional words or emotional facial expressions), thereby making the affective context task-relevant (Reynolds and Jeeves, 1978). Performance on the emotional go/nogo task correlates with performance on the traditional go/nogo task (in which no affective information is presented) thereby validating the emotional go/nogo task as a measure of response inhibition within an affective context (Schulz et al., 2007).

In adults, response inhibition appears to be better for negative compared to positive stimuli (Schulz et al., 2007; Chiu et al., 2008). This effect goes together with better emotion recognition for negative compared to positive stimuli (Schulz et al., 2007; Chiu et al., 2008). Within a large developmental sample (5- to 28-year-olds) a similar pattern was observed, with better overall response inhibition performance for negative stimuli, and this pattern did not differ with age (Tottenham et al., 2011). In this study different negative emotions were included, namely sadness, anger, and fear. Importantly, response inhibition performance appeared to be worst for the emotions for which emotion recognition was most difficult (sadness and anger). For fear, emotion recognition was best and response inhibition performance for fearful faces did not differ from response inhibition for positive (i.e., happy) stimuli, consistent with the notion that fearful faces are relatively easy to recognize (Tottenham et al., 2011).

Recently, studies have examined the influence of irrelevant emotions on response inhibition. In these studies, emotional stimuli were presented in the background and participants were instructed to respond to another dimension of the emotional stimulus (for example, the color or the direction of a stimulus). Importantly, all these tasks share the feature that participants do not have to focus on the emotional aspects of the stimuli for successful task performance. Within adult studies results are mixed, some studies show an effect of irrelevant emotions on response inhibition performance (Albert et al., 2010; Sagaspe et al., 2011), and other studies do not show an effect of irrelevant emotions on performance (Brown et al., 2012). In children and adolescents again mixed results were found, with two studies showing an effect of irrelevant emotions on response inhibition (Lamm et al., 2012; Cohen-Gilbert and Thomas, 2013), and another study not showing this effect (Todd et al., 2012).

Knowing how affective contexts influence response inhibition is of critical importance, since response inhibition is seldom required in a cold situation. Most often response inhibition is needed in hot affective situations, in which emotions play a role. Importantly, these emotions are not always relevant for the task at hand. Currently, it remains unknown whether relevant and irrelevant affective contexts have a similar or different influence on response inhibition. The goal of this study was therefore, to examine the influence of relevant and irrelevant affective context on response inhibition in children, adolescents and adults.

The present study is the first to directly compare the influence of both relevant and irrelevant emotions on response inhibition within the same participants of a developmental sample (covering mid-childhood to early adulthood). Participants performed two emotional response inhibition tasks, one in which emotion formed a relevant dimension of the task, and one in which emotion was irrelevant. In the first emotional go/nogo task emotion was a relevant dimension and participants had to respond when a given emotional facial expression was presented and inhibit responding when another emotional facial expression was presented (see Tottenham et al., 2011). In the second emotional go/nogo task emotion was an irrelevant dimension. In this task the same emotional faces were presented, but now faces were colored and participants had to respond to the color of the faces. A special feature of the latter task was the inclusion of intentional go/nogo trials (Brass and Haggard, 2007, 2008). The task therefore, involved three types of trials: go-trials, nogo-trials, and choice-trials (in which participants could freely decide between responding and inhibiting). These choice trials were added to the task to further examine the influence of irrelevant emotions on response inhibition in a free choice situation. Previous research in the field of free choice inhibition has been conducted in a neutral context (e.g., Brass and Haggard, 2007; Kühn et al., 2009). This research has indicated that free choice inhibition can be distinguished from externally driven inhibition on the basis of underlying neural mechanisms (Filevich et al., 2012). Here we aimed to examine the influence of affective context on free choice inhibition. In both tasks happy, fearful, and neutral faces were presented, since previous research has shown that within a developmental sample emotion recognition for these emotions is comparable (Tottenham et al., 2011).

We expected to observe a stronger effect of relevant compared to irrelevant emotions on response inhibition (Schulz et al., 2007; Tottenham et al., 2011; Brown et al., 2012; Todd et al., 2012). We expected this effect to be present across all age groups. Furthermore, we expected to find a developmental increase in inhibitory performance, and we aimed to examine whether this pattern was different depending on affective context (Tottenham et al., 2011). Finally, we aimed to test whether we would find a dip in response inhibition performance for happy stimuli in adolescents (Somerville et al., 2011) when emotion was a relevant or an irrelevant dimension.

## Materials and methods

### Participants

Ninety-four participants took part in the study. Participants were divided over five age groups; 18 6–7-year olds (*M* = 7.11, *SD* = 0.46, 8 females), 19 8–9-year-olds (*M* = 9.36, *SD* = 0.85, 8 females), 19 10–12-year-olds (*M* = 11.68, *SD* = 0.83, 10 females), 20 13–15-year-olds (*M* = 14.67, *SD* = 0.46, 9 females), and 18 18–25-year-olds (*M* = 21.04, *SD* = 2.16, 15 females). A χ^2^ test revealed no significant differences in gender distributions between age groups (*p* = 0.07). Children and adolescents were recruited from a primary and a secondary school in the Netherlands and informed consent was obtained from a primary caregiver. Adult participants were recruited from Leiden University and signed informed consent before participation in the experiment.

Participants completed the Raven Standard Progressive Matrices (Raven SPM) to obtain an estimate of their cognitive functioning (Raven et al., 1998). For one 6–7-year-old the Raven SPM was not completed. All estimated IQ scores were within the normal range. However, age groups differed in estimated IQ scores, *F*_(4, 92)_ = 6.00, *p* < 0.001. 13–15-year-olds had a significantly lower estimated IQ (*M* = 107.05, *SD* = 7.68) compared to the 6–7-year-olds (*M* = 124.35, *SD* = 14.77), the 8–9-year-olds (*M* = 120.84, *SD* = 11.16), and the 18–25-year-olds (*M* = 119.17, *SD* = 9.33) (all *p*'s < 0.02). 13–15-year-olds' estimated IQ did not differ significantly from the 10–12-year-olds (*M* = 116.53, *SD* = 14.17) (*p* = 0.09). Therefore, all analyses were performed twice, once without estimated IQ score and once with estimated IQ score added as a covariate. Since all results remained the same with estimated IQ score added as a covariate we here report only the results of the analyses without estimated IQ score as a covariate.

### Stimuli

Face stimuli were selected from the Radboud Faces Database (Langner et al., 2010). The selected set consisted of four adult males, four adult females, four child males, and four child females, all posing three different expressions (happy, fearful, and neutral), resulting in 48 stimuli in total. For the standard emotional go/nogo task face stimuli were transformed to gray scale images. For the colored emotional go/nogo task blue, purple, and orange color filters were placed over the gray scale images, resulting in blue, purple, and orange colored face images.

### Tasks

Participants performed two tasks; a standard emotional go/nogo task, in which emotion was a relevant dimension of the task, and a colored emotional go/nogo task in which emotion was an irrelevant dimension of the task.

#### Standard emotional go/nogo task

**Figure 1:**
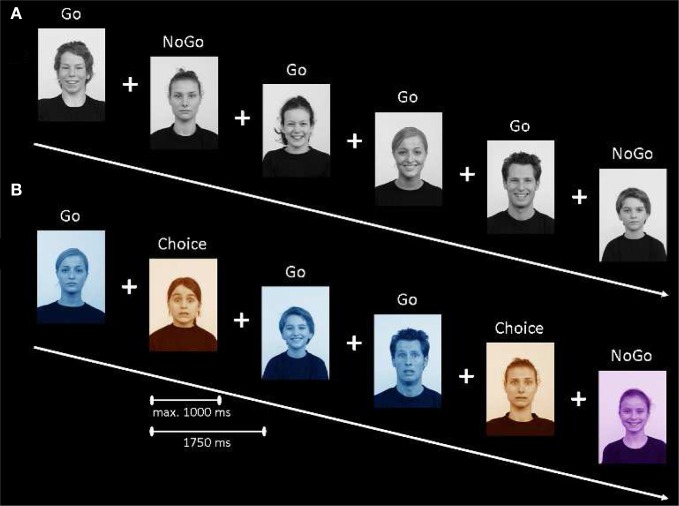
**(A)** Outline of the standard emotional go/nogo task. **(B)** Outline of the colored emotional go/nogo task. See text for details about the tasks.

The standard emotional go/nogo task was adapted from Tottenham et al. (2011). In this task participants had to respond by pressing a button when a given facial expression (e.g., happy) was presented and inhibit responding when another facial expression (e.g., neutral) was presented (see Figure 1A). In each task block emotional facial expressions (happy or fearful) were coupled with neutral facial expression, resulting in four different go/nogo blocks (happy/neutral, neutral/happy, fearful/neutral, and neutral/fearful, with the first emotion as the go-, and the second emotion as the nogo-stimulus). Participants were not instructed about the valence of the nogo-stimulus, but instead were instructed to inhibit responding when another facial expression than the go-stimulus was presented. Face stimuli were presented for a maximum duration of 1000 ms, and participants had to respond within that time window. When participants responded within the time window, the face stimulus disappeared from the screen and the remaining time was added to the presentation time of the fixation cross as a filler to keep the task duration equal across participants. The subsequent jitter (fixation cross) was presented for 750 ms.

Face stimuli were presented on a black background. Each task block consisted of 60 trials; 42 go-trials (to create a prepotency for responding) and 18 nogo-trials. Trials were presented in a pseudo-randomized order, such that there were never more than two consecutive nogo-trials. Task blocks were presented in a random order and before each task block participants were instructed which facial expression formed the go-stimulus.

#### Colored emotional go/nogo task

In the colored emotional go/nogo task emotion was an irrelevant dimension and participants were instructed to respond to the color of the faces (see Figure 1B). In this task there were three types of trials: go-trials, nogo-trials, and choice-trials. In the choice trial condition, participants could freely decide between responding and inhibiting. The task was explained to the participants as a catching game, in which they had to catch and inhibit catching members of colored teams. Participants were instructed that they had to catch the members of the blue team by pressing a button and had to inhibit catching members of the purple team. Participants were explained that people in the orange team were in disguise. Fifty percent of the orange team members actually belonged to the blue team and therefore, had to be caught, and the other 50% of the orange team members actually belonged to the purple team and therefore, should not be caught. Participants were instructed to freely decide and not use a strategy to decide when to respond or inhibit in the choice condition. The meaning (i.e., go, nogo, choice) of the colors was counterbalanced across participants, to control for possible a priori color preferences.

The temporal properties of the task were similar to the standard emotional go/nogo task; face stimuli were presented for a maximum duration of 1000 ms, and participants had to respond within that time window. When participants responded within the time window, the face stimulus disappeared from the screen and the remaining time was added to the presentation time of the fixation cross as a filler to keep the task duration equal across participants. The subsequent jitter (fixation cross) was presented for 750 ms.

Face stimuli were presented on a black background. In total there were 270 trials; 180 go-trials (to create a prepotency for responding), 30 nogo-trials, and 60 choice trials. Emotions (happy, fearful, and neutral) were equally distributed across conditions. Trials were presented in a pseudo-randomized order, such that there were never more than two consecutive nogo-trials or more than two consecutive choice-trials. The task was divided in two task blocks of 135 trials each.

### Procedure

All participants were tested in a laboratory or an empty classroom. Tasks were performed in a fixed order. First the colored emotional go/nogo task was performed, to ensure that emotion would be an irrelevant dimension in that task. Before each task participants were given instructions and performed a short practice block (18 trials for the colored task and 15 trials for the standard task). It was stressed that participants were not supposed to use a specific strategy to decide whether to act or inhibit in the choice condition of the colored task. Care was taken that all participants understood the instructions and were able to perform the tasks. Including instructions, the tasks took approximately 25 min. to complete. After completion of the tasks participants were asked whether they had used a specific strategy in the choice condition of the colored task. Finally, participants completed the Raven SPM.

### Data analysis plan

For both the standard and the colored emotional go/nogo task, the main variable of interest was the percentage of false alarms (i.e., failed inhibitions). Furthermore, we were interested in the percentage of correct responses on the go-trials and the reaction times for the correct go-trials to examine overall task performance. For the colored emotional go/nogo task an additional variable of interest was the percentage of nogo choices. These variables were added to repeated measures ANOVAs with Age group as a between subjects factor, and Emotion as within subjects factor, to examine task performance in the context of happy and fearful faces across development. For the standard emotional go/nogo task, Type (i.e., emotion as the go-stimulus vs. emotion as the nogo-stimulus) was added as an additional factor to the repeated measures ANOVAs to examine whether performance in the context of happy and fearful faces was the same independent of the emotional face being the go- or nogo-stimulus.

## Results

### Standard emotional go/nogo task

Four participants (one 6–7-year-old, two 8–9-year-olds, and one 13–15-year-old) were excluded from all analysis of this task because of misunderstanding of task instructions (i.e., reversal of go and nogo stimuli). Therefore, the final sample for this task consisted of 17 6–7-year olds (*M* = 7.09, *SD* = 0.46, 8 females), 17 8–9-year-olds (*M* = 9.37, *SD* = 0.88, 7 females), 19 10–12-year-olds (*M* = 11.68, *SD* = 0.83, 10 females), 19 13–15-year-olds (*M* = 14.71, *SD* = 0.41, 9 females), and 18 18–25-year-olds (*M* = 21.04, *SD* = 2.16, 15 females).

#### False alarms

**Figure 2:**
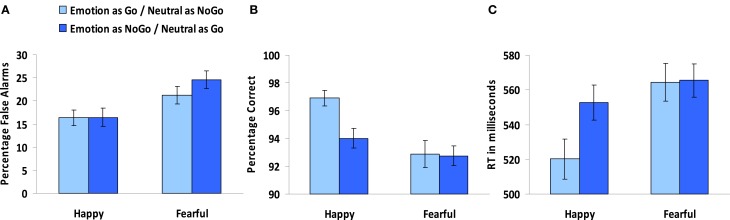
**Overall results of the standard emotional go/nogo task. (A)** Percentage of false alarms for the blocks with happy and fearful faces. **(B)** Percentage of correct responses for the blocks with happy and fearful faces. **(C)** RTs in milliseconds for the blocks with happy and fearful faces. Note that happy and fearful faces were presented in separate blocks, paired with neutral faces (see text for explanation).

To examine differences in the percentage of false alarms (i.e., failed inhibitions), an Age group (5) × Emotion (2: happy and fearful) × Type (2: emotion as go- or nogo-stimulus) repeated measures ANOVA was performed. A main effect of Emotion, *F*_(1, 85)_ = 50.89, *p* < 0.001, indicated that overall participants made more false alarms in blocks with fearful faces compared to blocks with happy faces (see Figure 2A). No main or interaction effects of Type were observed (all *p*'s > 0.08), indicating that performance did not differ based on the emotional face being the go- or nogo-stimulus.

**Figure 3:**
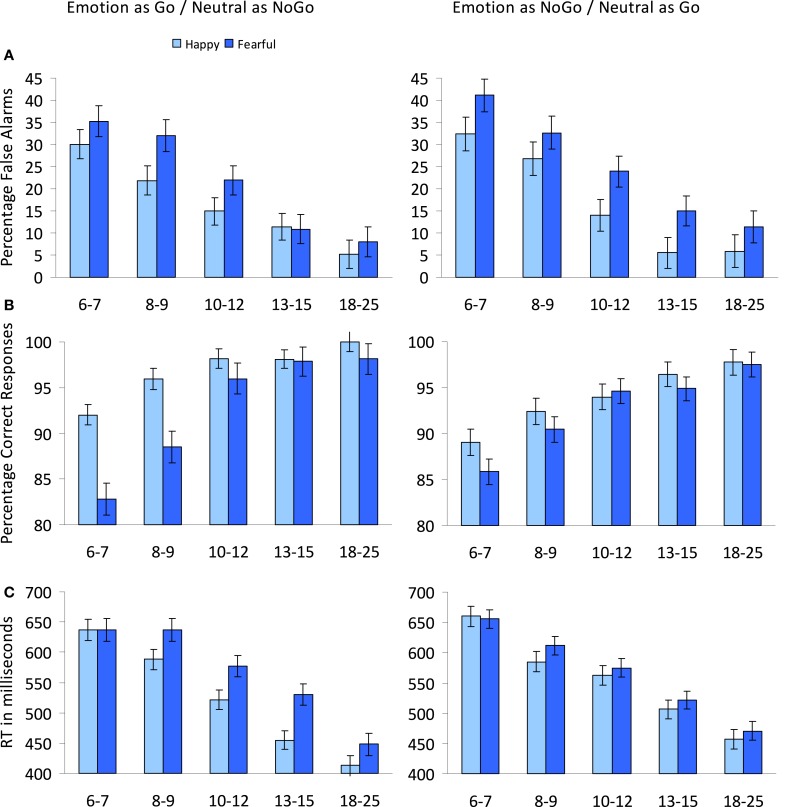
**Developmental differences on the standard emotional go/nogo task. (A)** Developmental decrease in the percentage false alarms for happy and fearful faces plotted separately for the blocks in which the emotional face was the go- vs. the nogo-stimulus. **(B)** Developmental increase in the percentage correct responses for happy and fearful faces plotted separately for the blocks in which the emotional face was the go- vs. the nogo-stimulus. **(C)** Developmental decrease in RTs to happy and fearful faces plotted separately for the blocks in which the emotional face was the go- vs. the nogo-stimulus. Note that happy and fearful faces were presented in separate blocks, paired with neutral faces (see text for explanation).

A main effect of Age group, *F*_(4, 85)_ = 14.72, *p* < 0.001, indicated that overall the percentage of false alarms decreased with age (see Figure 3A). *Post-hoc* Tukey tests showed that the 6–7-year-olds (*M* = 34.72, *SD* = 16.01) did not differ from the 8–9-year-olds (*M* = 28.35, *SD* = 16.76) (*p* = 0.58), but made significantly more false alarms compared to the other age groups (all *p*'s < 0.005). 8–9-year-olds did not differ from the 10–12-year-olds (*M* = 18.71, *SD* = 14.59) (*p* = 0.16), but made significantly more false alarms compared to the two oldest age groups (both *p*'s < 0.005). 10–12-year-olds, 13–15-year-olds (*M* = 10.60, *SD* = 5.28), and 18–25-year-olds (*M* = 7.64, *SD* = 5.54) did not differ significantly from each other (all *p*'s > 0.05). No interactions between Age group and Emotion or Type were observed (all *p's > 0.1).*

#### Percentage correct go-trials

To test for differences in the percentage of correct responses on the go-trials, an Age group (5) × Emotion (2: happy and fearful) × Type (2: emotion as go- or nogo-stimulus) repeated measures ANOVA was performed. A main effect of Emotion, *F*_(1, 85)_ = 23.22, *p* < 0.001, indicated that percentage correct go-trials was higher in blocks with happy faces compared to blocks with fearful faces. A main effect of Type, *F*_(1, 85)_ = 9.03, *p* < 0.005, indicated that percentage correct go-trials was higher in blocks in which the emotional face was the go-stimulus compared to blocks in which the neutral face was the go-stimulus. Finally as can be seen in Figure 2B, an Emotion × Type interaction, *F*_(1, 85)_ = 8.60, *p* < 0.005, indicated that better performance for blocks where the emotional face was the go-stimulus to relative to the nogo-stimulus, was driven by the blocks with happy faces. For the blocks with fearful faces it did not matter whether the emotional face was a go- or a nogo-stimulus (*p* = 0.84).

Overall, the percentage correct on the go-stimulus increased with age, *F*_(4,85)_ = 18.91, *p* < 0.001 (see Figure 3B). *Post-hoc* Tukey tests showed that the 6–7-year-olds (*M* = 87.43, *SD* = 6.45) made fewer correct responses compared to all other age groups (all *p*'s < 0.03). 8–9-year-olds (*M* = 91.84, *SD* = 4.40) did not differ significantly from the 10–12-year-olds (*M* = 95.68, *SD* = 2.91) (*p* = 0.058), but made significantly fewer correct responses compared to the two oldest age groups (both *p*'s < 0.01). 10–12-year-olds, 13–15-year-olds (*M* = 96.68, *SD* = 4.28), and 18–25-year-olds (*M* = 98.35, *SD* = 1.70) did not differ significantly from each other (all *p*'s > 0.3). An Emotion × Age group interaction, *F*_(4, 85)_ = 3.90, *p* < 0.01, showed that the difference between percentage correct for the blocks with happy faces compared to the blocks with fearful faces (independent of whether these were go- or nogo-stimuli) was larger for the youngest age groups (see Figure 3B).

#### Reaction times

Differences in reaction times to the go-stimuli were examined by performing an Age group (5) × Emotion (2: happy and fearful) × Type (2: emotion as go- or nogo-stimulus) repeated measures ANOVA. A main effect of Emotion, *F*_(1, 85)_ = 51.02, *p* < 0.001, showed that reaction times were faster for the blocks with happy faces compared to the blocks with fearful faces. A main effect of Type, *F*_(1, 85)_ = 12.30, *p* < 0.005, showed that reaction times were faster for the blocks in which the emotional face was the go-stimulus compared to block in which the neutral face was the go-stimulus. As can be seen in Figure 2C an Emotion × Type interaction, *F*_(1, 85)_ = 11.86, *p* < 0.005, indicated that the effect of faster reaction times when the emotional face was the go-stimulus was driven by the block with happy faces, for the blocks with fearful faces conditions did not differ (*p* = 0.61).

Overall, reaction times to the go-stimuli decreased with age, *F*_(4, 85)_ = 29.28, *p* < 0.001 (see Figure 3C). *Post-hoc* Tukey tests showed that the 6–7-year-olds (*M* = 647, *SD* = 56) did not differ from the 8–9-year-olds (*M* = 605, *SD* = 70) (*p* = 0.28), but were significantly slower compared to the other age groups (all *p*'s < 0.001). 8–9-year-olds did not differ from the 10–12-year-olds (*M* = 559, *SD* = 52) (*p* = 0.17), but were significantly slower compared to the two oldest age groups (both *p*'s < 0.001). 10–12-year-olds were slower compared to both the 13–15-year-olds (*M* = 504, *SD* = 65) (*p* < 0.05) and the 18–25-year-olds (*M* = 447, *SD* = 63) (*p* < 0.001). The 13–15-year-olds were also significantly slower compared to the 18–25-year-olds (*p* < 0.05). An Emotion × Age group interaction, *F*_(4, 85)_ = 4.40, *p* < 0.005, indicated that the emotion effect of faster response times for blocks with happy faces than for blocks with fearful faces was driven by the four oldest age groups, whereas the youngest age groups did not differ in reaction times to emotions (see Figure 3C). A Type × Age group interaction, *F*_(4, 85)_ = 2.88, *p* < 0.05, indicated that the type effect of faster reaction times for the blocks in which the emotional face was the go-stimulus was reversed for the 8–9-year-olds (see Figure 3C).

Together, these results indicate that task relevant emotions influence task performance such that participants are more accurate and faster for happy faces than for fearful faces, and that this effect is strongest for the younger age groups. Overall, the results suggest that the blocks with fearful faces were more difficult compared to the blocks with happy faces.

### Colored emotional go/nogo task

#### False alarms

**Figure 4:**
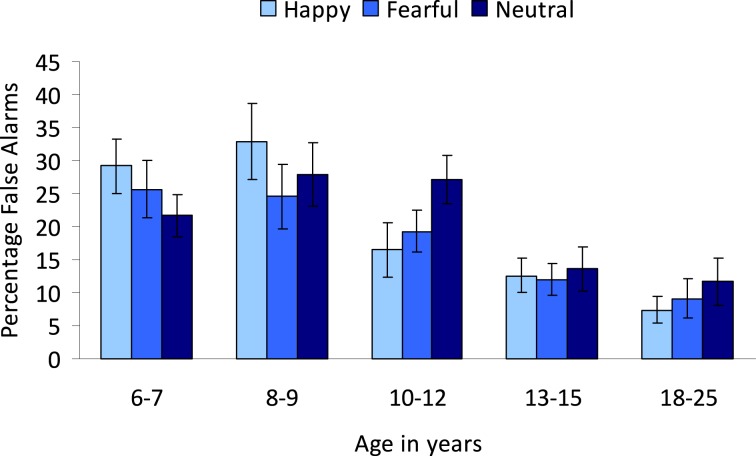
**Percentage false alarms on the colored emotional go/nogo task**. Participants were instructed to respond to colors and emotion was an irrelevant dimension.

In order to examine the influence of irrelevant emotions on false alarms, an Age group (5) × Emotion (3: neutral, happy, and fearful) repeated measures ANOVA was performed for the nogo-trials. No main effect of Emotion was observed (*p* > 0.4). Overall, the percentage of false alarms decreased with age, *F*_(4, 89)_ = 7.26, *p* < 0.001 (see Figure 4). *Post-hoc* Tukey tests showed that the 6–7-year-olds (*M* = 25.56, *SD* = 12.68) did not differ from the 8–9-year-olds (*M* = 28.25, *SD* = 20.07) (*p* = 0.96) and both groups made more false alarms compared to the two oldest groups (13–15-year-olds: *M* = 12.83, *SD* = 9.44, 18–25-year-olds: *M* = 9.44, *SD* = 9.85) (all *p*'s < 0.05). The 10–12-year-olds (*M* = 21.05, *SD* = 10.89) did not differ significantly from any group (all *p*'s > 0.06).

#### Percentage correct go-trials

**Figure 5:**
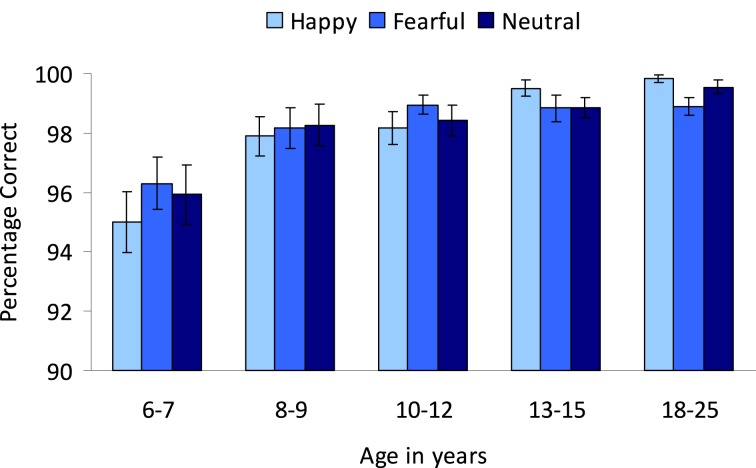
**Percentage correct on the colored emotional go/nogo task**. Participants were instructed to respond to colors and emotion was an irrelevant dimension.

To examine whether the irrelevant emotion of the go-stimulus influenced correct responding, an Age group (5) × Emotion (3: neutral, happy, and fearful) repeated measures ANOVA was performed. No main effect of Emotion was observed (*p* > 0.7). Overall, correct responding to the go-trials increased with age, *F*_(4, 89)_ = 7.58, *p* < 0.001 (see Figure 5). *Post-hoc* Tukey tests showed that the 6–7-year-olds (*M* = 95.74, *SD* = 3.59) made fewer correct responses to the go-stimuli compared to all other age groups (8–9-year-olds: *M* = 98.10, *SD* = 2.79, 10–12-year-olds: *M* = 98.51, *SD* = 1.57, 13–15-year-olds: *M* = 99.06, *SD* = 1.33, 18–25-year-olds: *M* = 99.41, *SD* = 0.75) (all *p*'s < 0.05). The other age groups did not differ significantly from each other (all *p*'s > 0.3).

#### Nogo choices

**Figure 6:**
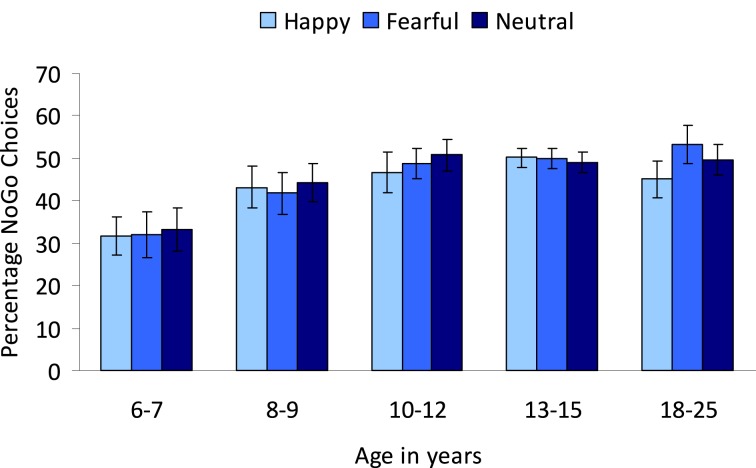
**Percentage nogo choices on the colored emotional go/nogo task**. Participants were instructed to respond to colors and emotion was an irrelevant dimension.

To further examine the influence of irrelevant emotions on action and inhibition, an Age group (5) × Emotion (3: neutral, happy, and fearful) repeated measures ANOVA for the choice trials was performed. No main effect of Emotion was observed (*p* > 0.2). Overall, the percentage of nogo-choices for choice trials increased with age, *F*_(4, 84)_ = 4.64, *p* < 0.005 (see Figure 6). *Post-hoc* Tukey tests showed that the 6–7-year-olds (*M* = 32.31, *SD* = 20.10) did not differ significantly from the 8–9-year-olds (*M* = 43.07, *SD* = 19.70) (*p* = 0.16), but made significantly fewer nogo-choices compared to the oldest three age groups (10–12-year-olds: *M* = 48.86, *SD* = 14.84, 13–15-year-olds: *M* = 49.75, *SD* = 7.24, 18–25-year-olds: *M* = 49.44, *SD* = 8.71) (all *p*'s < 0.01). The oldest four age groups did not significantly differ in the percentage of nogo-choices (all *p*'s > 0.5).

#### Reaction times

To examine whether irrelevant emotions influenced reaction times differently depending on condition (go vs. choice), an Age group (5) × Condition (2: go and choice) × Emotion (3: neutral, happy, and fearful) repeated measures ANOVA was performed. A main effect of Condition, *F*_(1, 89)_ = 220.41, *p* < 0.001, indicated that making an intentional decision to act took more time compared to acting in response to an external go-stimulus.

**Figure 7:**
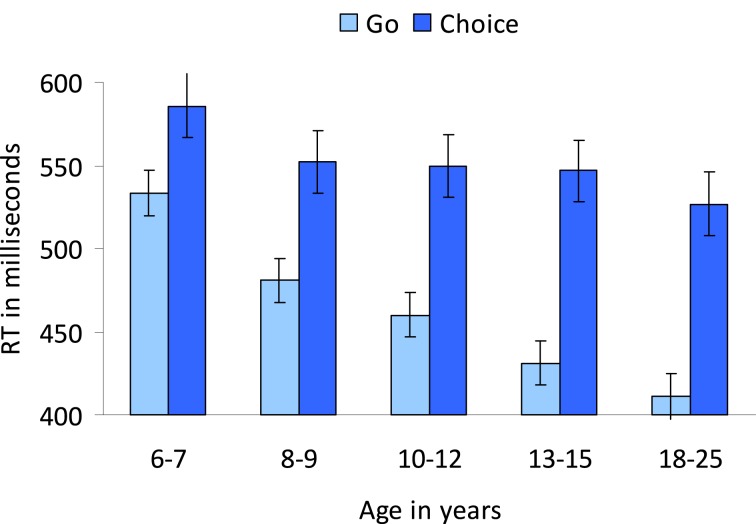
**Developmental differences in RTs for the go- and choice-conditions**. Participants were instructed to respond to colors and emotion was an irrelevant dimension.

Overall, reaction times decreased with age, *F*_(4, 89)_ = 5.04, *p* < 0.005 (see Figure 7). *Post-hoc* Tukey tests showed that the 6–7-year-olds were significantly slower compared to the two oldest age groups (both *p*'s < 0.05). The other age-groups did not differ significantly from each other (all *p*'s > 0.08).

As indicated by a Condition × Age group interaction, *F*_(4, 89)_ = 4.25, *p* < 0.005, developmental differences in reaction times were more pronounced in the choice compared to go condition (see Figure 7).

Together, these results show an increase in performance with age. Irrelevant emotions had no effect on performance. Also in the choice condition, decisions to act or inhibit were not influenced by irrelevant emotions.

## Discussion

The present study examined the influence of relevant and irrelevant emotions on response inhibition across child and adolescent development. For this means participants performed two emotional go/nogo tasks, one in which emotion formed a relevant dimension and one in which emotion was an irrelevant dimension. The latter task also involved a condition in which participants could make free choices between responding and inhibiting. Here we will discuss the findings of the present study related to (1) the main effects of emotion on response inhibition, (2) the linear age-related increase in response inhibition performance within an affective context, and (3) the influence of irrelevant affective context in a free-choice situation.

### Emotion effects

In the standard emotional go/nogo task relevant emotions influenced response inhibition. Overall, the blocks with fearful faces were more difficult compared to the blocks with happy faces. This finding could indicate that emotion recognition was easier in blocks with happy compared to fearful faces. Previous studies with the emotional go/nogo task have shown better performance (i.e., fewer false alarms) for negative compared to positive emotions (Schulz et al., 2007; Chiu et al., 2008; Tottenham et al., 2011). It should be noted that this reversed effect of better performance for negative emotions was found in the context of sad or anger facial expressions (Schulz et al., 2007; Tottenham et al., 2011) and in the context of generally negative words (Chiu et al., 2008). The one study also including fearful faces did not show a difference in performance between fearful and happy faces (Tottenham et al., 2011). Thus, we reasoned based on prior studies that fearful faces and happy faces should not differ in demands on emotional recognition. Yet, the finding that performance was *worse* for fearful faces than for happy faces contrasts with these earlier studies.

Our observation of decreased performance in the blocks with fearful faces could be explained by literature showing negative effects of threatening stimuli on cognitive control in general (Lindstrom and Bohlin, 2012) and response inhibition in particular (Hartikainen et al., 2012; Lindstrom and Bohlin, 2012). Fearful faces are an indicator of possible threat and therefore, might have a similar negative effect on response inhibition as threatening stimuli. Future studies should examine this effect of emotion valence *vis-a-vis* demands on emotion recognition in more detail.

One of the specific questions we aimed to study was whether the effect of emotions on inhibition was different for relevant and irrelevant affective contexts. In line with our expectations we found strong effects of relevant emotions on response inhibition, but no effects of irrelevant emotions on response inhibition. This is in agreement with prior studies showing small or no effects of irrelevant emotions on response inhibition (Albert et al., 2010; Sagaspe et al., 2011; Brown et al., 2012; Lamm et al., 2012; Todd et al., 2012). This strengthens the hypothesis that differences in demands on emotion recognition may be a stronger indicator of inhibition performance than the presence of an emotion *per se*. Future studies should unravel whether it is truly the effect of an emotion that influences inhibition, or if other stimuli which differ in recognition demands would also result in inhibitory differences.

### Developmental effects

Previous research has reported two interesting effects with respect to the development of response inhibition. First, several studies have reported a developmental increase in inhibitory control (van der molen, 2000; Durston et al., 2002; Tottenham et al., 2011; Cohen-Gilbert and Thomas, 2013), a finding which was supported in this study. Second, a prior study showed a dip in response inhibition performance for happy faces during mid-adolescence (Somerville et al., 2011). This dip has been interpreted as an increased tendency to approach appetitive stimuli within adolescence, which is supported by the observation of increased activation in striatal reward areas when seeing those positive stimuli (Somerville et al., 2011). We aimed to test this hypothesis in more detail in a large sample, but we could not replicate this finding, neither when emotion was a relevant dimension, nor when emotion was an irrelevant dimension of the task. Instead, we observed a linear increase in task performance independent of the influence of relevant and irrelevant emotions. This is in line with the findings of Tottenham et al. (2011) who also did not find an adolescent dip in performance on the standard emotional go/nogo task and findings of Cohen-Gilbert and Thomas (2013) who did not find an adolescent dip in performance on an emotional go/nogo task with irrelevant emotions. One possibility is that this effect is dependent on specific faces which were used or task instructions. The non-linear development of sensitivity to emotions should be studied in more detail in future experiments.

In agreement with Tottenham et al. (2011) we showed a stable effect of emotions on response inhibition across development, such that inhibition was more difficult for fearful faces than for happy faces, especially when emotions were relevant for the task. When emotions were irrelevant, we showed a small enhancement of happy faces on response inhibition, but response inhibition for fearful faces was comparable to response inhibition for neutral faces. In contrast, Cohen-Gilbert and Thomas (2013) showed decreased performance in the context of negative compared to neutral and positive irrelevant emotions across development. However, in this study pictures from the International Affective Picture System (IAPS; Lang et al., 2008) were used as irrelevant background stimuli. Negative pictures from the IAPS might be more salient in capturing attention compared to the fearful faces used in the present study, which could explain why we did not find a negative effect of irrelevant fearful faces in the present study (see also Hartikainen et al., 2012; Lindstrom and Bohlin, 2012). Previous research has suggested that seeing emotional faces involves more emotion recognition, whereas seeing IAPS stimuli involves more emotion evocation (Britton et al., 2006), resulting in more arousal. Therefore, arousal instead of valence *per se* might be the driving factor through which affective stimuli influence response inhibition.

### Free choice

In the colored emotional go/nogo task free choice-trials were added to further examine the effect of irrelevant emotions on response inhibition. One could expect to observe a stronger effect of irrelevant emotions in a free choice situation, given that participants can use the affective context to base their decisions on (Brass et al., 2013). However, decisions to act or inhibit were not made on the basis of affective context. Brass et al. (2013) argued that in free choice situation social context can be an important motivator for behavior. Moreover, free choice experiments with an affective, social, or motivational context are more ecologically valid compared to the standard free choice experiments in which participants can choose between arbitrary response options (Brass et al., 2013). Here, however, we did not show an effect of irrelevant affective context on choice behavior. Future studies should address whether a more salient affective, social, or motivational context has a stronger effect on behavior.

There were also developmental differences in deciding between acting and inhibiting on the choice-trials. Young children in general chose to inhibit on a fewer percentage of trials than adults, which can reflect the same underlying mechanism as standard stimulus-driven inhibition (van der molen, 2000; Bunge et al., 2002; Durston et al., 2002; Luna et al., 2010). Thus, even in a choice context it was more difficult for them to choose to inhibit. Second, all participants were slower on choice trials than on go trials, consistent with prior studies on free choice (Kühn et al., 2009). However, there was no developmental difference in reaction times on free choice trials, whereas there was a steep developmental decrease in reaction time to standard go trials. The latter finding is consistently reported in the developmental literature (Tamnes et al., 2012), but the absence of age differences in choice trials suggests that the deliberation time of choice vs. no choice puts additional demands on reaction time in adolescents and adults. But most importantly, this deliberation time was not dependent on irrelevant affective context. An interesting question for future research will be to add a free choice condition to the task where affective context is relevant.

### Limitations

The present study has a number of limitations. First, the standard and the colored emotional go/nogo tasks were explained in a slightly different manner. The colored emotional go/nogo task was explained in a game-framework, whereas the standard emotional go/nogo task was explained with standard instructions. This might have influenced how participants have experienced the tasks. If the colored emotional go/nogo task was experienced more as a game, this might have been more engaging for the younger participants. However, it is not very likely that this difference in framing had an effect on how emotion influenced task performance.

Second, the free choice condition was only presented in the emotional go/nogo task with irrelevant emotions. Therefore, we could not examine whether relevant affective contexts, in contrast to irrelevant affective contexts, would have an effect on choices to act or inhibit. A fruitful direction for future research would be to add such a free choice condition to an emotional go/nogo task with relevant emotions, to further unravel the effects of relevant affective context on response inhibition.

Third, the color of the emotional faces differed between tasks. In the standard emotional go/nogo task emotional faces were presented in gray scale, whereas in the colored emotional go/nogo task emotional faces were presented in color. Therefore, the relevance of emotion is confounded with the absence or presence of color. Future studies should examine whether the different influence of relevant vs. irrelevant emotion remains present, when color remains constant.

Fourth, the present study was a behavioral study. Therefore, we could not examine the underlying mechanisms of the influence of relevant and irrelevant emotions on response inhibition. Future studies comparing the influence of relevant and irrelevant emotions on response inhibition should include psychophysiological measures to examine these mechanisms. Previous studies focusing on the effect of irrelevant emotions on response inhibition did not all observe effects on the behavioral level (e.g., Brown et al., 2012). However, all studies showed an effect of irrelevant emotions on the neural level, indicative of increased effort when applying response inhibition within an affective context (Albert et al., 2010; Brown et al., 2012). One open question is whether this effect is equally strong for relevant and irrelevant emotions.

### Summary and conclusion

Affective or social contexts can interact with cognitive control, especially during childhood and adolescence (Blakemore and Robbins, 2012). The present study set out to directly compare the influence of relevant and irrelevant emotions on response inhibition across development. We show that with increasing age there is a linear increase in response inhibition performance within affective contexts. In this large cross-sectional sample we have found no evidence of a previously observed mid-pubertal dip in affective response inhibition (Somerville et al., 2011).

Furthermore, our results indicate that across development relevant emotions have a stronger effect on response inhibition compared to irrelevant emotions, and this effect was stronger in young children. In a free choice situation people did not base their decisions on irrelevant affective context. An interesting question for future research would be to incorporate a free choice condition in a response inhibition task with relevant affective context to further elucidate the influence of affective context on cognitive control.

### Conflict of interest statement

The authors declare that the research was conducted in the absence of any commercial or financial relationships that could be construed as a potential conflict of interest.
